# Comparison of outcome of unilateral locking plate and dual plating in the treatment of bicondylar tibial plateau fractures

**DOI:** 10.1186/s13018-014-0062-y

**Published:** 2014-07-20

**Authors:** Meng-Hsuan Lee, Chien-Jen Hsu, Kai-Cheng Lin, Jenn-Huei Renn

**Affiliations:** 1 Department of Orthpaedics, Kaohsiung Veterans General Hospital, 386 Ta-Chung 1st Rd, Kaohsiung 81346, Taiwan

## Abstract

**Background:**

Tibial plateau fracture (TPF) includes different fracture patterns with varied degrees of articular depression and displacement. Many kinds of fixators, including newly designed plate with locking screws, were applied to treat these complicated fractures. We intended to follow up the surgical outcomes of (1) unilateral locking plate, (2) classic dual plates, or (3) hybrid dual plates for TPF.

**Materials and methods:**

We retrospectively reviewed 76 patients with TPF, Schatzker types V and VI, who we operated from June 2006 to May 2009 in our institute. Excluding patients who expired due to other medical conditions and without complete follow-up, 45 patients were sorted out in this series. The scheme of surgical intervention was designed by visiting staff, and 15 patients, as group I, were treated with unilateral locking plate. The other 19 patients, as group II, were treated with classic dual plates. The residual 11 patients, as group III, were treated with hybrid dual plates (one lateral approach locking compression plate (LCP) + medial anti-gliding plate). All patients were under periodic F/U at about 6 weeks interval for at least 18 months postoperatively.

**Results:**

In group I, 13 cases achieved solid bony union without obvious traumatic OA change, limitation of ROM, or malalignment. In groups II and III, 15 and 10 patients reached the same goal, respectively. By analysis of the recorded parameters with statistical software (SPSS 12.0), there were five parameters with significant difference, including Schatzker classification, operation time, staged treatment or not, hospitalization period, and hardware impingement.

**Conclusions:**

There was no significant statistical difference of union rate between these three groups in our series. Based on our clinical follow-up, several key points were emphasized: (1) Soft tissue problems should be kept in mind, and usage of locking plate can reduce the discomfort of hardware impingement effectively. (2) The single lateral approach technique for TPF with locking plate results in less operation time and shorter hospitalization period. (3) If the medial buttress cannot be established by reduction of the lateral fracture, then open reduction of the medial side is necessary and buttresses the medial fragment by dual plates.

## Introduction

Tibial plateau fractures (TPFs) have a complicated intra-articular fracture pattern, representing approximately 1.2% of all fractures [[1]]. Surgical treatment for high-energy displaced bicondylar fractures of the tibia plateau remains a challenge for most surgeons. According to the Schatzker classification, types V and VI are complex fractures often associated with soft tissue injury, a high risk of wound complications, difficulty in reduction, and further sufficient fixation for stabilization. However, the ideal fixation method is not yet clear, and treatment options include screws, an external fixator, hybrid external fixation [[2],[3]], limited internal fixation combined with a tensioned wire [[4]], classic dual buttress plates, a unilateral periarticular locking plate, and hybrid dual plates (combination of locking plate and buttress plate).

In highly unstable bicondylar fractures, open reduction and internal fixation (ORIF) with dual plating has been biomechanically proven as an effective method for stabilization after reduction of both fracture fragments and articular surfaces. However, fixation with dual plating requires extensive soft tissue dissection and thus increases the risks of wound complications. There are many journals using a unilateral periarticular locking plate in the treatment of bicondylar TPFs with a lower risk of soft tissue damage and surgical site infection. They reported that both stabilization methods are equally effective [[5]-[7]].

The purpose of this retrospective study was to analyze our experience with consecutive high-energy tibial plateau fractures, Schatzker type V or type VI, involving a bicondylar component, which were managed using a unilateral periarticular locking plate, classic dual buttress plates, or hybrid dual plates (combination of locking plate and buttress plate).

## Materials and methods

**Figure 1:**
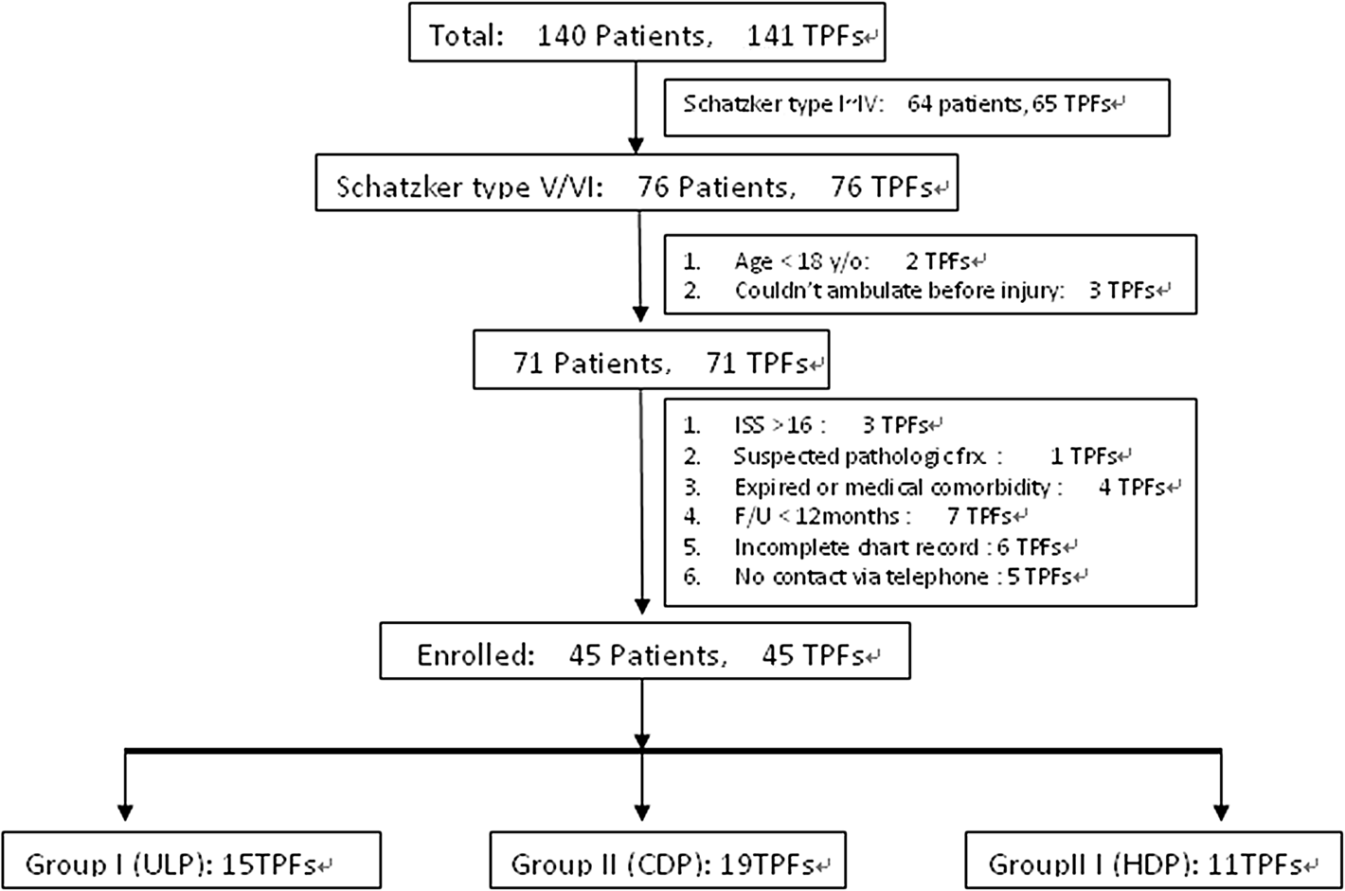
Number of patients in this study.

We retrospectively reviewed the medical records of patients with high-energy bicondylar TPFs, diagnosed in our institute between June 2006 and May 2009. Patients were identified from the register of the Accident and Emergency Department or theater logs, and data were accumulated from case notes, operative records, and radiographs. A total of 140 patients with 141 TPFs were treated. Of these fractures, 65 were found to be Schatzker I ~ IV and another 76 consecutive patients were classified as having bicondylar TPFs of Schatzker type V/VI. The inclusion criteria of this study were the presence of bicondylar TPFs Schatzker type V/VI, patients aged over 18 years, and the ability to walk without assistance before injury. Polytrauma patients with TPFs with an injury severity score (ISS) >16 [[8]] and patients with bilateral plateau fractures were excluded from this study. Cases with suspected pathologic fracture, in which the patient expired, or in which the patient has other medical conditions resulting in failure in the evaluation of functional outcome (The Western Ontario and McMaster Universities Osteoarthritis Index (WOMAC)); those with a period of follow-up less than 18 months; patients with incomplete chart records; and those who could not be contacted via telephone were also excluded. Figure 1 shows the cases in this study. All fractures were classified into a unilateral locking plate (group I), classic dual buttress plates (group II), or hybrid dual plates (group III), composed of a lateral approach periarticular locking compression plate (LCP) + a medial anti-gliding buttress plate.

Forty-five patients with complex tibial plateau fractures were enrolled, and the study group consisted of 21 males (47%) and 24 females (53%) with an average age of 51.68 years (range 18 ~ 83 years). The mechanism of injury was a motor vehicle accident in 38 patients, and the others were falls from height. All patients had anteroposterior and lateral view radiographs at our emergency department initially, and a CT scan was performed if the fracture pattern was difficult to classify. There were 25 Schatzker type V (55.5%) and 20 Schatzker type VI (44.4%) fractures. The soft tissue condition was the most crucial item on our preoperative planning for the timing of the operation and the choice of surgical equipment. Twelve patients (26.7%) received staged therapy, with an average of a 5.4-day delay (range 3 ~ 9 days) due to open fracture or extreme swelling of soft tissue (external skeletal fixation first, then shifted to an internal fixator). Prophylactic antibiotics with first-generation cephalosporin, cefazolin, were administered intravenously in all patients and were prescribed as necessary for at least the first day. Other than the routine prophylactic antibiotics, the patients with open fracture may have received a combined aminoglycoside, gentamycin, and the agents were subsequently replaced according to the culture results.

Surgery was performed under general or spinal anesthesia. Patients were set up in the supine position on the operating table, with a knee flexion of 15° ~ 30°. A tourniquet was used to diminish blood loss and was deflated for no more than 2 h. During the operation, the fracture reduction was visualized via an image intensifier, and arthrotomy was always performed to check the congruity of the articular surface. Temporary fixation with Kirschner wire or an interfragmentary screw was a helpful technique, and bone substitute or allogenous bone grafts were applied to elevate the osseous gaps. A Hemo-Vac drain tube was placed in the operation wound, and the wound was closed primarily.

All patients were followed up according to the postoperative protocol shown below. In the initial 6 weeks postoperatively, patients were educated to avoid weight bearing, and in the following 6 weeks, partial weight-bearing ambulation with a walking frame was prescribed. Full weight-bearing walking was then allowed once solid union was observed on plain film. We arranged periodic follow-ups at 6-week intervals to take radiographs and record clinical function. Three criteria were used to be sure about union on plain films: (1) bridging callus between fragments, (2) obliteration of previous fracture gap, and (3) no further displacement of fracture fragment including articular surface after full weight-bearing ambulation during serial follow-up images. The standard anteroposterior (AP) radiographs of the injured knee should be obtained immediately postoperatively and at the time of fracture healing by two experienced observers. A positive value was applied to angles representing a valgus deformity, while negative values were applied to a varus deformity. According to previous clinical articles, malunion with malreduction or malalignment is defined as change of alignment of (1) intra-articular step-off over 2 mm or (2) angulation over 5° in AP or lateral view radiographs [[9]].

The chi-square test was used for comparison of categorical variables between the three groups, and the one-way independent ANOVA test was applied for continuous variables, such as age, operation time, blood loss, and functional outcome. Post-hoc analysis was further used for determining which groups differed. All data were assessed via SPSS 12.0 (SPSS, Inc., Chicago, IL, USA), and a *p* value lower than 0.05 was accepted as statistically significant in all analyses [[10]].

## Results

A total of 76 patients were identified as having bicondylar TPFs Schatzker type V or VI, and 45 patients met the criteria for this study. All had at least 18 months follow-up from June 2006 to May 2009. Of the 45 patients, 15 were fixed with a unilateral locking plate (group I), 19 were fixed with classic dual buttress plates (group II), and 11 with hybrid dual plates (group III), which consist of a combination of lateral approach periarticular LCP and a medial anti-gliding buttress plate. No significant differences existed regarding gender ratio, mean age, injured limb, and mechanism of trauma between the three groups. The descriptive data of these patients are summarized in Table 1.

**Table 1:** **Demographic data of the patients with bicondylar tibial plateau fractures (*N*** **= 45)**

	**Group I (ULP)**	**Group II (CDP)**	**Group III (HDP)**
Case number	15	19	11
Male/female ratio	5/10	11/8	5/6
Mean age^a^ (years) (range)	49.1 ± 13.4 (18 ~ 83)	53.6 ± 14.8 (18 ~ 76)	51.8 ± 17.0 (22 ~ 81)
Injured limb (right/left)	9/6	9/10	4/7
Trauma mechanism (TA/fall)	13/2	16/3	9/2
Schatzker classification^£^ (V/VI)	8/7	13/6	4/7

^a^Examined by one-way independent ANOVA test. The others were examined by the chi-square test. ^£^*p* < 0.05, statistically significant difference between the compared groups, *ULP* unilateral locking plate, *CDP* classic dual plate, *HDP* hybrid dual plate, *TA* traffic accident.

Table 2 reports the perioperative parameters and main functional outcomes. The listed data were available for all 45 patients during surgery, the period of hospitalization, and the serial follow-ups after discharge. There were statistically significant differences in perioperative parameters, including the duration of surgery, the period of hospitalization, and whether staged management was needed between the three groups. Advanced post-hoc analysis was required to identify the differences between the three groups.

**Table 2:** **Perioperative parameters and postoperative functional scores in each group of bicondylar tibial plateau fractures (total*N*** **= 45)**

	**Group I (ULP)**	**Group II (CDP)**	**Group III (HDP)**
	***N*** **= 15**	***N*** **= 19**	***N*** **= 11**
Operation time^£^ (min)	76.6 ± 14.73 (50 ~ 145)	101.4 ± 18.23 (60 ~ 160)	92.8 ± 17.96 (55 ~ 150)
Blood loss (ml)	69.3 ± 14.36 (30 ~ 180)	100.1 ± 24.63 (40 ~ 350)	82.7 ± 24.31(40 ~ 250)
Staged Tx (ESF → IF)^a,£^	20% (3/15)	21.1% (4/19)	45.5% (5/11)
Hospitalization period^£^ (days)	9.3 ± 4.27 (5 ~ 26)	15.6 ± 8.71 (7 ~ 51)	14.8 ± 5.52 (6 ~ 43)
Post-OP alignment (degree)	87.9 ± 6.4 (76 ~ 99)	87.0 ± 3.9 (75 ~ 93.4)	85.1 ± 7.3 (74 ~ 95)
Union rate^a^	86.7% (13/15)	78.9% (15/19)	90.9% (10/11)
Functional outcome (WOMAC 0 ~ 96)	36.5 ± 5.88 (7 ~ 66)	34.1 ± 4.91 (6 ~ 61)	32.8 ± 5.02 (7 ~ 59)
Pain 0 ~ 20	5.4 ± 2.31 (0 ~ 12)	4.9 ± 2.06 (0 ~ 13)	4.5 ± 1.8 (1 ~ 11)
Stiffness 0 ~ 8	4.8 ± 1.68 (0 ~ 7)	4.3 ± 1.21 (0 ~ 6)	4.1 ± 1.83 (0 ~ 6)
Physical function 0 ~ 68	26.3 ± 5.74 (4 ~ 50)	24.9 ± 4.82 (4 ~ 49)	24.2 ± 6.44 (3 ~ 42)

^a^Examined by the chi-square test. The others were examined by the one-way independent ANOVA test. ^£^*p* < 0.05, statistically significant difference between the compared groups. *ULP* unilateral locking plate, *CDP* classic dual plate, *HDP* hybrid dual plate, *Tx* treatment, *ESF* external skeletal fixation, *IF* internal fixation.

Overall, five patients (11%) faced non-union after 1 year of follow-up, and the average union rate was between 78% and 91% in the three groups. Comparisons of the functional outcome, using the WOMAC score, disclosed no significant differences between the three groups, and further evaluation of subgroups, such as pain, stiffness, or physical function, demonstrated no differences. The mean WOMAC score was 34.6 (range 6 ~ 66), and the mean score for pain, stiffness, and physical function was 4.97 (range 0 ~ 13), 4.41 (range 0 ~ 7), and 25.2 (range 3 ~ 50), respectively.

We recorded several kinds of complications (Table 3) in this study: infection, knee stiffness (<90° flexion), posttraumatic arthritis, malunion, non-union, hardware impingement, re-fracture, and implant failure. No significant differences existed regarding the above items, with the exception of the ratio of hardware impingement, which required a secondary operation to remove the implants. All patients with infection were managed with local wound care and oral antibiotics, but three cases developed deep infections, which were treated with repeat irrigation, debridement, intravenous antibiotics, and follow-up at the Infection Clinic. In our study, no malreduction was measured on the first postoperative radiographs. However, after 12 months of follow-up, secondary loss of reduction was revealed in three patients in group I (unilateral locking plate (ULP)), three patients in group II (classic dual plates (CDP)), and one patient in group III (hybrid dual plates (HDP)). Re-fracture or implant failure was found in three patients due to another trauma or poor compliance with partial weight-bearing ambulation, and these patients underwent further surgery for re-osteosynthesis.

**Table 3:** Complications after surgical intervention for bicondylar tibial plateau fractures

	**Group I (ULP)**	**Group II (CDP)**	**Group III (HDP)**	***p*** **value**
	***N*** **= 15**	***N*** **= 19**	***N*** **= 11**	
Infection				
Deep	1	1	1	0.792
Cellulitis	2	2	1	0.328
Stiffness (<90° flexion)	2	3	2	0.651
Posttraumatic arthritis	3	3	1	0.267
Malunion				
Angulation (>5°)	1	0	0	0.515
Joint depression (>2 mm)	2	3	1	0.449
Non-union	2	4	1	0.373
Hardware impingement	1	5	1	0.021*
Re-fracture	1	1	0	0.603
Implant failure	0	1	0	0.584

All examined by the chi-square test. *ULP* unilateral locking plate, *CDP* classic dual plate, *HDP* hybrid dual plate. *p < 0.05, statistically significant difference between the compared groups.

**Figure 2:**
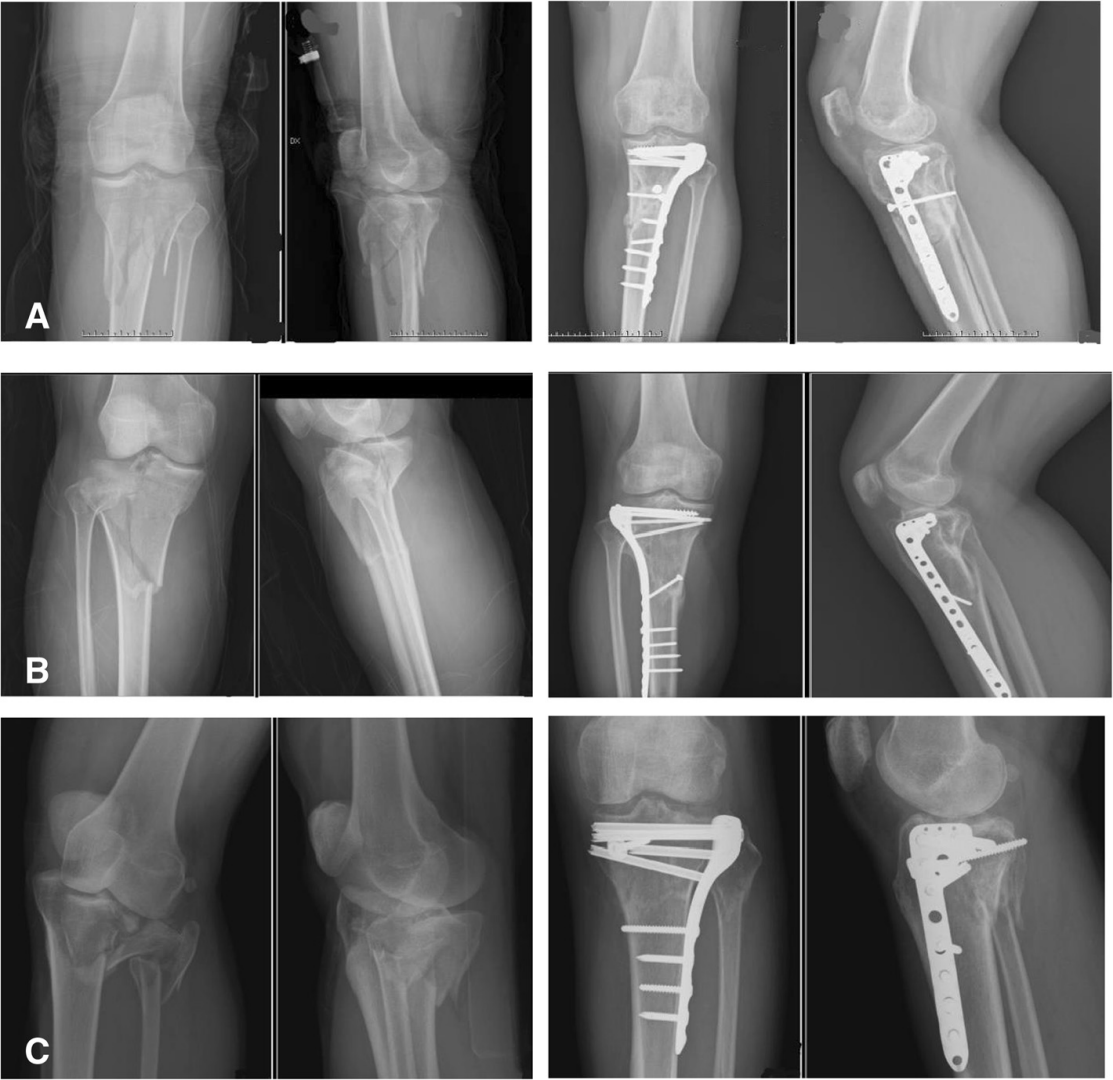
**Illustrative cases of group I, treated with unilateral locking plate (ULP). (A)** A 39-year-old female sustained a left knee tibial plateau fracture (TPF), Schatzker classification type VI, and she was subsequently treated with a unilateral locking plate as shown. **(B)** An 18-year-old female sustained right knee Schatzker type VI TPF after being in a motor vehicle accident. She subsequently received ORIF with ULP. **(C)** A 51-year-old male sustained left knee Schatzker type V TPF with dislocation after being in a crash accident. He then received ORIF with ULP.

**Figure 3:**
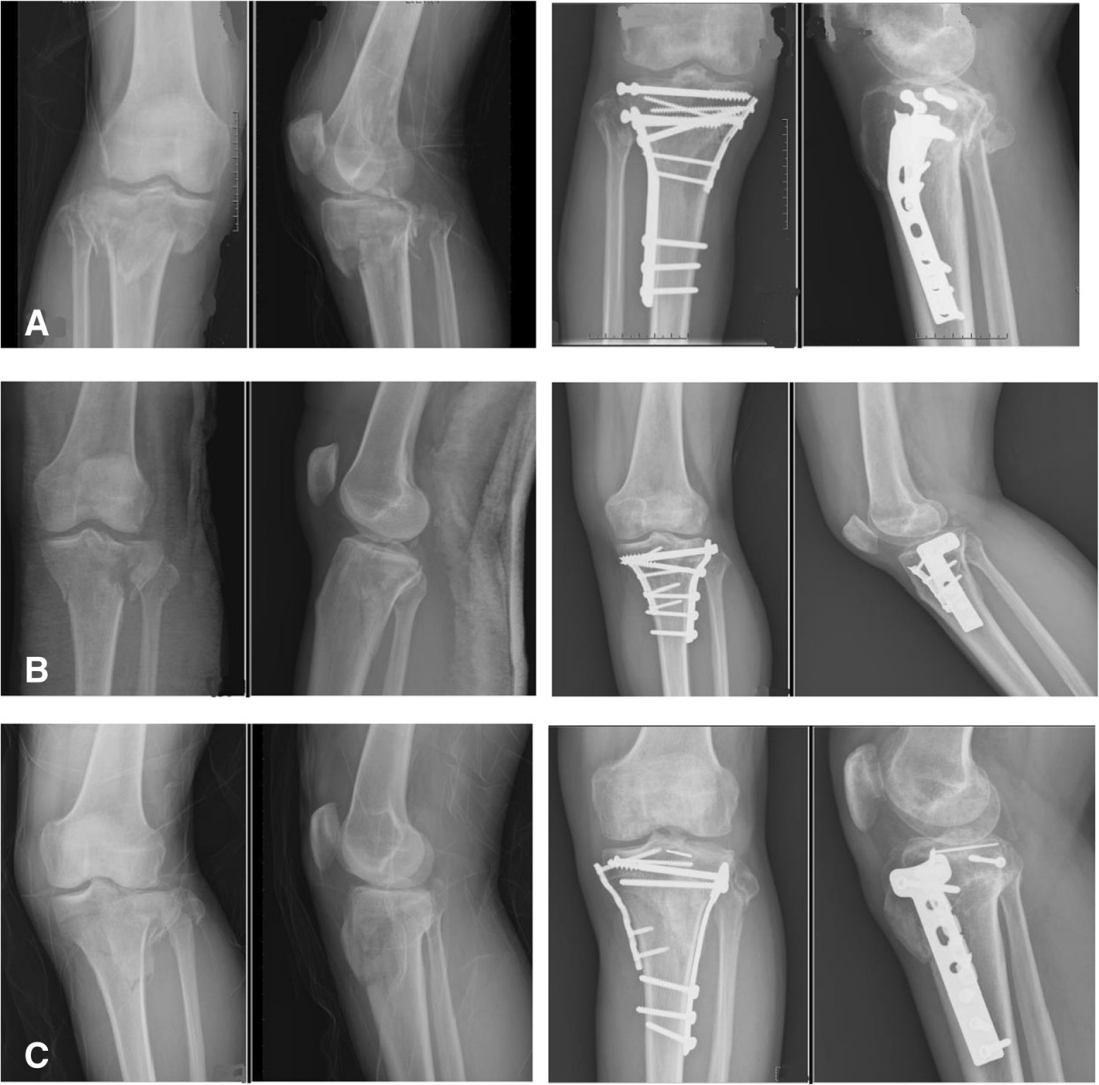
**Illustrative cases of group II, treated with classic dual plates (CDP). (A)** A 49-year-old man sustained right knee TPF, Schatzker classification type V, after being in a motor vehicle crash. ORIF with CDP was demonstrated. **(B)** A 47-year-old woman sustained left knee Schatzker type V TPF after being in a falling accident. She subsequently received ORIF with CDP. **(C)** A 60-year-old man sustained left knee Schatzker type VI TPF. He received ORIF with CDP thereafter.

**Figure 4:**
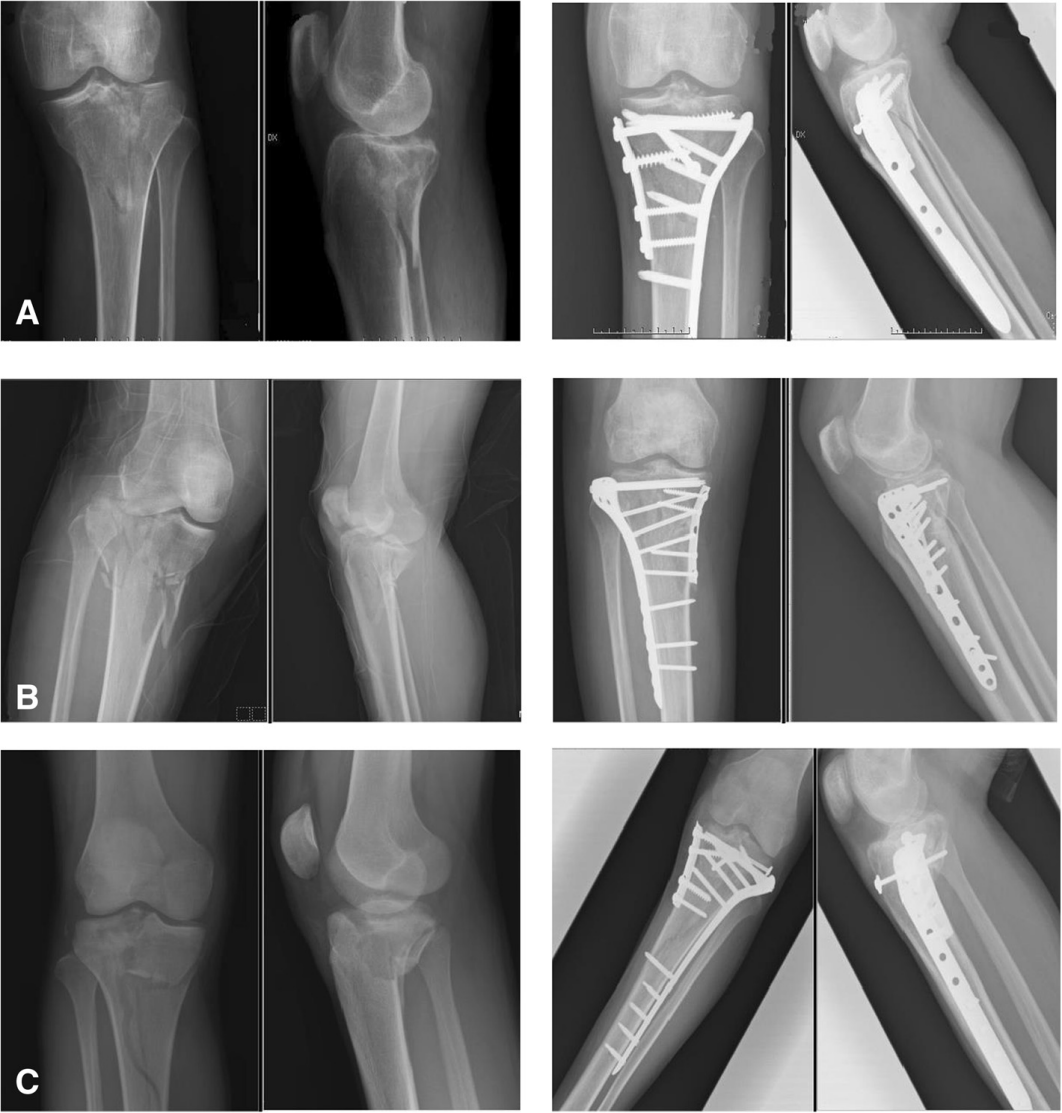
**Illustrative cases of group III, treated with hybrid dual plates (HDP). (A)** A 76-year-old man suffered from left knee TPF, Schatzker classification type VI, after a slithering accident, and he subsequently received ORIF with HDP. **(B)** A 43-year-old woman sustained right knee Schatzker type V TPF after being in a motor vehicle crash. She received ORIF with HDP thereafter. **(C)** A 19-year-old man suffered from right knee Schatzker type VI TPF in a car crash. ORIF with HDP was demonstrated.

The items exhibiting a statistically significant difference were identified, for example, operation time and complication of hardware impingement, and all are listed in Table 4. Post-hoc tests with the Scheffe method were applied to identify which groups differed. The result demonstrated the following: (1) There was a significantly different ratio for Schatzker classification type V or VI between group II (CDP) and group III (HDP). (2) In some cases of open fracture or obvious swelling of soft tissue and impending compartment syndrome, staged treatment, initially external skeletal fixation then shifted to an internal fixator, was arranged. Group III (HDP) had a markedly higher percentage of these cases than group I (ULP) or group II (CDP). (3) In group II, the average operation time was 101.4 ± 18.23 min and the hospitalization period was 15.6 ± 8.71 days. This is a longer operation time and a longer hospitalization period than for group I and group III. (4) Five people in group II felt discomfort over the lateral aspect of the injured knee, especially while walking too far or climbing stairs, and they requested us to remove it after bony union of the tibial plateau fracture. The ratio was higher than in group I and group III. Illustrative cases are shown in Figures 2, 3, and 4.

**Table 4:** Post-hoc analysis of the three groups fixed with different implants

	**ULP vs. CDP**	**ULP vs. HDP**	**CDP vs. HDP**
Schatzker classification^a^ (V/VI)	ns	ns	*p* < 0.05
Operation time (min)	*p* < 0.05	*p* < 0.05	ns
Staged Tx (ESF → IF)^a^	ns	*p* < 0.05	*p* < 0.05
Hospitalization period (days)	*p* < 0.05	*p* < 0.05	ns
Hardware impingement^a^	*p* < 0.05	ns	*p* < 0.05

^a^Examined by the chi-square test. The others were examined by the one-way independent ANOVA test. *p* < 0.05, statistically significant difference. *ns* not significant, *ULP* unilateral locking plate, *CDP* classic dual plate, *HDP* hybrid dual plate, *Tx* treatment, *ESF* external skeletal fixation, *IF* internal fixation.

## Discussion

TPFs were classified using the Schatzker classification system, which distinguishes low-energy split/depression fractures from higher energy bicondylar fractures (Schatzker type V/VI). For most surgeons, bicondylar TPFs remain an arduous challenge because the knee is a biomechanically complex joint and these fracture patterns are often combined with trauma to the surrounding soft tissue. To achieve the most satisfactory outcome, a well-designed preoperative surgical strategy with minimal unnecessary soft tissue injury must be designed. Over the past years, a number of treatment modalities have been developed, such as simple skin traction, cast immobilization, external skeletal fixation, and open reduction and internal fixation with different implants. In this article, conventional 3.5-mm buttress plates, dynamic compression plates (DCPs), 1/3 tubular plates for anti-gliding, and pre-contour periarticular locking compression plates (LCPs) were applied for fixation.

The goals of operative treatment for TPFs were anatomic reduction, especially in restoration of articular congruity, stable fixation for early rehabilitation, and avoidance of complications, particularly infection and non-union. In our study, we included a series of 45 bicondylar TPFs, Schatzker type V or VI, which were all followed up for at least 18 months. We found a significantly different ratio of Schatzker type V/VI between group II (CDP) and group III (HDP); this may be because the fracture pattern of Schatzker type VI is bicondylar metaphyseal involvement with diaphyseal extension, and the pre-contour periarticular locking compression plate offers more advantages than classic buttress plates for fixation via the minimally invasive plate osteosynthesis (MIPO) technique. Turning to the surgical procedure, we applied the single pre-contour periarticular locking plate over the lateral compartment via the MIPO technique in group I, thus avoiding medial periosteal striping [[11],[12]]. This method reduced the operation time effectively, and patients sustained less discomfort resulting from surgical manipulation of an already injured soft tissue envelope. Furthermore, a shorter hospitalization period and lower cost were also noted in this group. Kenneth et al. [[13]] reported 38 patients with complex tibial plateau fracture treated with LISS plating system and concluded that it provides stable fixation of complex bicondylar TPFs allowing early range of knee motion with favorable clinical results.

Barei et al. [[14],[15]] and Ali et al. [[16]] reported that single lateral locked plating may not be as effective as dual plating in managing bicondylar tibial plateau fractures, and lots of previous studies have demonstrated a postoperative malreduction rate of 15% ~ 23% using the LISS plating system [[5],[11]]; other studies have reached opposite conclusions [[5],[7]]. A similar rate of postoperative malalignment of about 20% (3/15) was noted in group I (ULP), and there was no statistically significant difference between the other groups, group II 15.8% and group III 9.1%, respectively. On further reflection, all of these three postoperative malalignment cases which had posterior-medial compartment were noted in group I. This may be not fixed by unilateral locking plate method. Barei et al. concluded that although laterally applied fixed-angle plate devices have demonstrated excellent early results for the management of the high-energy bicondylar TPF, alternate or adjunctive methods, such as supplemental medial exposures and implants, may need to be considered to completely stabilize this injury pattern. Higgins et al. [[6]] performed a biomechanical study and concluded that dual-plate fixation allows less subsidence in this bicondylar tibial plateau cadaveric model when compared to isolated locked lateral plates. Barei et al. [[9]] reported 83 AO/OTA type-41-C3 bicondylar TPFs treated with medial and lateral plate fixation through two exposures. They concluded that medial and lateral plate stabilization of comminuted bicondylar TPFs through medial and lateral surgical approaches is a useful treatment method to achieve well functional outcome though some cases still have residual dysfunction.

Our study was a rare study comparing conventional dual plates with hybrid dual plates and showed similar functional outcome when compared with a locking plate. Restoration of mechanical stability with reliable fixation methods leads to early rehabilitation, a greater range of motion, and an acceptable union rate, which result in a better functional outcome. In this series, the 45 cases in the three groups were all evaluated using the WOMAC questionnaire. The average bone union rate was 78.9% ~ 90.9%, and no differences were noted between the groups, in accordance with Russell et al. [[17]]. Papagelopoulos et al. [[18]] also emphasized the importance of rigid fixation with early motion to avoid intra-articular adhesion, and these three fixation methods all provided enough mechanical stability to allow for postoperative rehabilitation protocols.

The high-energy traumatic mechanism is thought to be a major cause of poor results in tibial plateau fractures, due to the associated soft tissue damage and the comminuted severity of articular congruity. To treat these injuries, different methods have been proposed, and several perioperative-postoperative complications have been presented. Overall, we had an incidence of deep infection of 5.3% ~ 9.1%, malunion of 9.1% ~ 20%, and a non-union rate of 9.1%–21.1%, which were similar results to previous studies. Regarding posttraumatic arthritis of 9.1% ~ 20%, the average follow-up period was 13.6 months in our study and may need more time to analyze the impact of this fracture episode to posttraumatic arthritis. Interestingly, the complication of hardware impingement exhibited significantly more cases in group II (CDP), which may have resulted from the original design of the buttress plates.

There are some limitations of this study. First of all, this is a retrospective study, so selection bias can be a limitation. Surgical intervention with open reduction and internal fixation was performed in all cases, and the patients were sorted into three groups according to the implants used for fixation. By the surgeons' subjective judgment, the kind of implant chosen for fixation was in light of the fracture pattern, soft tissue condition, patients' insurance, etc. Second, we have medium case numbers in each group. The analytical power can be reduced in this case number. Third, TPFs are commonly associated with intra-articular soft tissue injury, such as ligamentous or meniscal damage [[14]], which may influence the postoperative functional outcome, and this was not discussed in our study.

## Conclusion

There were no significant statistical differences in union rate or functional outcome between the three groups in our series. Based on our clinical follow-up, several key points should be emphasized: (1) Soft tissue problems should be always kept in mind, and usage of a locking plate can reduce the discomfort of hardware impingement effectively. (2) The single lateral approach technique for TPF with a pre-contour locking plate reduces the soft tissue dissection, operation time, and hospitalization period. (3) If the medial buttress cannot be established by reduction of the lateral fracture, then open reduction of the medial side is necessary, and the medial fragment should be buttressed with a dual plate. (4) A trend of a higher postoperative malalignment rate over the posterior-medial compartment was noted in group I, but more cases and further evaluation are needed for verification. (5) There were still many limitations in this study, and further prospective randomized control trials should be established.

## Competing interests

The authors declare that they have no competing interests.

## Authors' contributions

MHL, CJH, and KCL designed the main framework. MHL carried out the data collection, interpretation of data, and statistical analysis, and drafted the manuscript. KCL and JHR provided critical revision of the manuscript for intellectual content and performed the final check for this manuscript. All authors read and approved the final manuscript.
